# Strategies to enhance venous thromboprophylaxis in hospitalized medical patients (SENTRY): a pilot cluster randomized trial

**DOI:** 10.1186/1748-5908-8-1

**Published:** 2013-01-02

**Authors:** Menaka Pai, Nancy S Lloyd, Ji Cheng, Lehana Thabane, Frederick A Spencer, Deborah J Cook, R Brian Haynes, Holger J Schünemann, James D Douketis

**Affiliations:** 1 Department of Medicine, McMaster University, Hamilton, ON, Canada; 2 Department of Clinical Epidemiology and Biostatistics, McMaster University, Hamilton, ON, Canada; 3 Hamilton Regional Laboratory Medicine Program, Hamilton, ON, Canada; 4 Biostatistics Unit, St Joseph’s Healthcare—Hamilton, Hamilton, ON, Canada; 5 Centre for Evaluation of Medicines, St Joseph’s Healthcare—Hamilton, Hamilton, ON, Canada

**Keywords:** Thromboprophylaxis, Medical patients, Anticoagulants, Venous thromboembolism, Cluster randomization, Standard orders

## Abstract

**Background:**

Venous thromboembolism (VTE) is a common preventable cause of mortality in hospitalized medical patients. Despite rigorous randomized trials generating strong recommendations for anticoagulant use to prevent VTE, nearly 40% of medical patients receive inappropriate thromboprophylaxis. Knowledge-translation strategies are needed to bridge this gap.

**Methods:**

We conducted a 16-week pilot cluster randomized controlled trial (RCT) to determine the proportion of medical patients that were appropriately managed for thromboprophylaxis (according to the American College of Chest Physician guidelines) within 24 hours of admission, through the use of a multicomponent knowledge-translation intervention. Our primary goal was to determine the feasibility of conducting this study on a larger scale. The intervention comprised clinician education, a paper-based VTE risk assessment algorithm, printed physicians’ orders, and audit and feedback sessions. Medical wards at six hospitals (representing clusters) in Ontario, Canada were included; three were randomized to the multicomponent intervention and three to usual care (*i.e.*, no active strategies for thromboprophylaxis in place). Blinding was not used.

**Results:**

A total of 2,611 patients (1,154 in the intervention and 1,457 in the control group) were eligible and included in the analysis. This multicomponent intervention did not lead to a significant difference in appropriate VTE prophylaxis rates between intervention and control hospitals (appropriate management rate odds ratio = 0.80; 95% confidence interval: 0.50, 1.28; *p* = 0.36; intra-class correlation coefficient: 0.022), and thus was not considered feasible. Major barriers to effective knowledge translation were poor attendance by clinical staff at education and feedback sessions, difficulty locating preprinted orders, and lack of involvement by clinical and administrative leaders**.** We identified several factors that may increase uptake of a VTE prophylaxis strategy, including local champions, support from clinical and administrative leaders, mandatory use, and a simple, clinically relevant risk assessment tool.

**Conclusions:**

Hospitals allocated to our multicomponent intervention did not have a higher rate of medical inpatients appropriately managed for thromboprophylaxis than did hospitals that were not allocated to this strategy.

## Background

Venous thromboembolism (VTE) is a common preventable cause of mortality in hospitalized patients [1-3]. Approximately 60% of symptomatic VTE occurs in medical patients, and recent hospitalization for medical illness accounts for 25% of all community-diagnosed VTE [1-3]. Not surprisingly, multiple agencies have identified thromboprophylaxis as a patient safety priority [4,5], and it has become a key requirement for hospital accreditation [6-8]. Thromboprophylaxis has been shown in multiple well-designed trials to be effective, safe, cost-saving, and easy to administer [9]. Despite these compelling considerations, nearly 40% of at-risk medical patients receive inappropriate thromboprophylaxis [10-14].

Prior knowledge-translation (KT) interventions using “high-tech” strategies, such as electronic alerts, computerized decision support systems, and computerized order entry, have improved prophylaxis rates [15-18]. One study of 2,506 hospitalized patients showed that electronic alerts reduced the risk of VTE by 41% after 90 days [17]. The results of these studies are encouraging but are not applicable to hospitals without the required computer infrastructure. A single-center “low-tech” study, with potentially more generalizable results, used education sessions, a paper-based decision support tool, and audit and feedback sessions in hospitalized medical and surgical patients [19]. This intervention increased the proportion of at-risk patients who received thromboprophylaxis from 58% to 93% over three years. The single-center BEHAVE study found that multidisciplinary education sessions, verbal reminders, daily charting of prophylaxis, and weekly feedback to individual physicians increased the median patient-days of thromboprophylaxis from 60 to 91 days in a medical-surgical intensive care unit [20]. While the impact of these low-tech interventions is also promising, understanding whether their impact is generalizable requires evaluation in diverse hospital settings with medical patients who are at greatest risk of inappropriate thromboprophylaxis.

To lay the groundwork for our trial, we conducted questionnaires and interviews of healthcare providers to assess perceptions about the importance of thromboprophylaxis, barriers to optimizing prophylaxis, and the potential success and feasibility of interventions to optimize prophylaxis [21,22]. While perceived as important, not all healthcare providers recognized that thromboprophylaxis was underused in medical patients. Our pilot studies suggested that a potentially successful and feasible intervention would be comprehensive and would be comprised of healthcare provider education, screening and risk-stratifying all patients, and preprinted orders. This concept was supported by published systematic reviews [23-25] and thus, we created a KT intervention that included these components.

The ***S***trategies to ***En***hance Venous ***T***h***r***omboembolism Proph***y***laxis in Hospitalized Medical Patients (SENTRY) pilot trial was designed to determine if a low-tech paper-based strategy could be feasibly implemented and if it could improve *appropriate* thromboprophylaxis in hospitalized medical patients. Appropriate and inappropriate thromboprophylaxis were defined *a priori* according to the criteria outlined in Table 1. Furthermore, to be deemed “appropriate”, we required the correct administration or the correct nonadministration of thromboprophylaxis (as per the American College of Chest Physicians [ACCP] guidelines) to occur within 24 hours of admission to the internal medicine service. The primary objective was to determine the feasibility of implementing the multicomponent SENTRY intervention. We also sought to determine if hospitals allocated to our intervention had a higher proportion of hospitalized medical patients appropriately managed for thromboprophylaxis within 24 hours of admission than hospitals not allocated to this strategy and if it minimized errors of commission (*i.e.*, administering prophylaxis when unnecessary) and errors of omission (*i.e.*, not administering prophylaxis when necessary).

**Table 1:** Definition of appropriate thromboprophylaxis

		**Did patient receive prophylaxis?**		
		**YES – pharmacologic prophylaxis**	**YES – mechanical prophylaxis**	**NO**
**Did the patient have thrombosis risk factors?**^**a**^	**YES – and bleeding risk factor**^**b**^	Error of commission	Appropriate prophylaxis (appropriate receipt)	Error of omission
	**YES – and no bleeding risk factors**	Appropriate prophylaxis (appropriate receipt)	Error of omission	Error of omission
	**NO**	Error of commission	Error of commission	Appropriate prophylaxis (appropriate nonreceipt)

^a^Thrombosis risk factors: congestive heart failure, chronic obstructive pulmonary disease, interstitial lung disease, acute ischemic stroke, active cancer, sepsis, acute neurological disease, inflammatory bowel disease, prior venous thromboembolism. ^b^Bleeding risk factors: acute bleed, at risk for bleeding, recent bleed, coagulopathy, thrombocytopenia.

## Methods

This study received approval from the Institutional Research Ethics Board at each participating site. As the study involved a minimal/low-risk intervention, each Board determined that it could be conducted without informed consent from patients or surrogates.

### Setting and participants

SENTRY was a 16-week cluster randomized controlled trial (RCT) conducted in the medical wards of six hospitals in Ontario, Canada from January to April 2009. Hospitals were the unit of randomization, and each hospital represented a cluster. Eligible hospitals were chosen based on geographic convenience, the availability of a physician willing to serve as the local principal investigator, and the lack of a formal institutional strategy to improve thromboprophylaxis. To ensure balance between the intervention and control groups, we used a stratified randomization strategy, based on the Ontario Ministry of Health and Longterm Care’s Group A/B/C classification of hospitals. Each group included one hospital from Group A (academic hospitals), one hospital from Group B (community hospitals having greater than 100 beds), and one hospital from Group C (community hospitals having fewer than 100 beds). Both Group A hospitals had a consultative thromboembolism service. None of the hospitals used medical patient admission order sets. Randomization of clusters, using a random number table, was concealed, with a 1:1 allocation sequence within strata. The allocation sequence was generated by the study statistician.

We enrolled all patients ≥18 years of age admitted to the service of general internal medicine during the study period. Patients admitted under cardiology, intensive care, or other medical subspecialties were not included. Patients were excluded if they were receiving therapeutic anticoagulation or had a length of stay less than 24 hours. For patients admitted more than once, only the first admission was included. Data on age, sex, admission diagnosis, length of stay, risk factors for thrombosis, risk factors for bleeding, the use of mechanical prophylaxis (intermittent pneumatic compression devices or anti-embolic stockings), and anticoagulant prophylaxis (unfractionated or low-molecular-weight heparin) were recorded from the hospital chart, using a standardized data collection form. Throughout this study, we made the assumption that if prophylaxis was ordered in the hospital chart, it was actually received by the patient.

### Intervention

**Figure 1:**
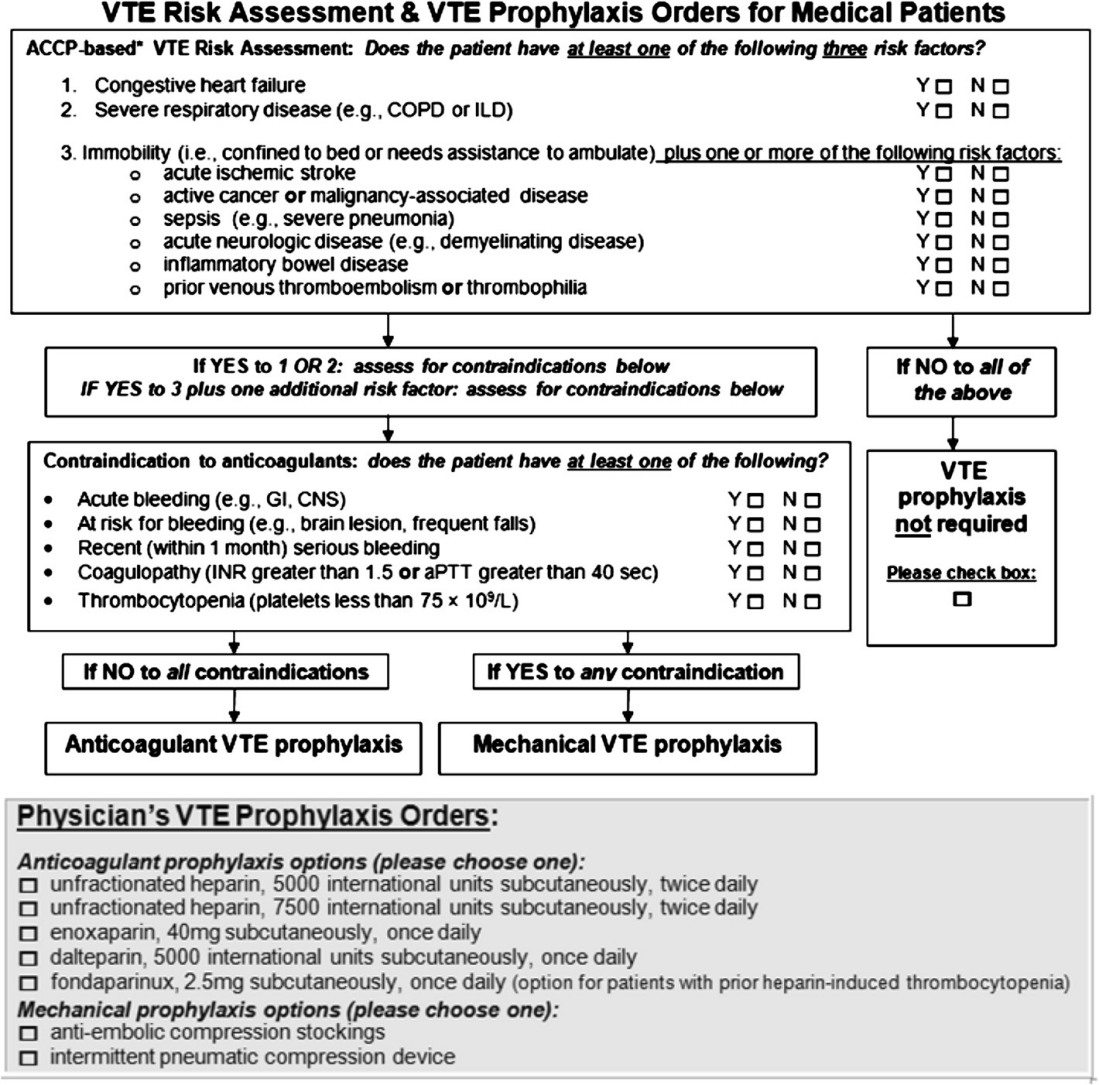
**Standardized VTE risk assessment and physician order form.** Legend: ACCP = American College of Chest Physicians, VTE = venous thromboembolism, COPD = chronic obstructive pulmonary disease, ILD = interstitial lung disease, GI = gastrointestinal, CNS = central nervous system, INR = international normalized ratio, aPTT = activated partial thromboplastin time.

This study was not blinded. Medical wards in the control group received “usual care”, which we defined as having no active or passive KT strategies to improve thromboprophylaxis in place. Medical wards in the intervention group received a multicomponent intervention consisting of the following:

1. Education sessions: Distribution of posters; a pamphlet describing current VTE practice patterns and the need to optimize thromboprophylaxis; and bimonthly educational sessions for ward physicians (and house staff, if applicable), nurses, and pharmacists. Pamphlets were distributed in paper format and via electronic mail to individuals who could not attend the sessions.

2. Standardized VTE risk assessment algorithm and physicians’ orders: (see Figure 1) These paper-based forms, modeled after the 8^th^ edition of the ACCP Evidence-Based Clinical Practice Guidelines, were available on internal medicine wards during the study period [4]. Local principal investigators (PIs) were asked to ensure that clinical staff were aware of the forms and to encourage their completion for eligible patients.

3. Audit and feedback: Real-time chart audits of eligible patients were done to determine whether patients were appropriately managed for thromboprophylaxis within 24 hours of admission. The entire health record during the relevant admission was searched to corroborate thrombosis and bleeding risk factors. All audits were done by one of two data management assistants, and data entry was validated by the study coordinator (NL) and two research assistants. Performance-based feedback sessions were done at 4, 12, and 16 weeks to relay results to clinical staff. Aggregate feedback was provided verbally and in a written handout at the sessions. The handout was also distributed in paper format and via electronic mail to individuals who could not attend the sessions.

### Outcomes

We defined the primary study outcome—feasibility—*a priori* according to the proportion of eligible medical patients who received appropriate prophylaxis, based on the criteria shown in Table 1. The intervention was considered definitely feasible, possibly feasible, or not feasible if the difference in the proportion of at-risk patients receiving appropriate prophylaxis in the intervention hospitals versus the usual care hospitals was >25%, 10–25%, and <10%, respectively. The secondary outcomes of this study were as follows:

1. The percentage of patients appropriately managed for thromboprophylaxis within 24 hours of admission to the internal medicine service (*i.e.*, administering prophylaxis when necessary, and not administering prophylaxis when unnecessary, as per the ACCP guidelines)

2. The percentage of patients subject to errors of commission within 24 hours of admission to the internal medicine service (*i.e.*, administering prophylaxis when unnecessary as per the ACCP guidelines)

3. The percentage of patients subject to errors of omission within 24 hours of admission to the internal medicine service (*i.e.*, not administering prophylaxis when necessary as per the ACCP guidelines)

The recommendations of the 8^th^ ACCP Evidence-Based Clinical Practice Guidelines on Antithrombotic Therapy were used to determine if prophylaxis was appropriate or not. As per the guidelines, thromboprophylaxis is indicated for patients with congestive heart failure, acute respiratory disease, and immobility plus one or more additional risk factors for VTE (acute ischemic stroke, active cancer, sepsis, acute neurologic disease, inflammatory bowel disease, previous VTE, or thrombophilia). Patients were considered to be immobile if there was a physician order for bed rest or if chart notes indicated the patient could not ambulate without support. Recommended pharmacologic prophylaxis regimens include low-dose unfractionated heparin (UFH) (5000 U twice daily or thrice daily) or low-molecular-weight heparin (LMWH) at manufacturers’ suggested prophylactic doses. Recommended mechanical prophylaxis options include elastic stockings or intermittent pneumatic compression devices. The “receipt of appropriate thromboprophylaxis” was defined as a physician’s order for the correct type and dose of thromboprophylaxis prescribed within the first 24 hours of admission, taking into account the individual patient’s thrombosis and bleeding risks. The rate of compliance, the duration of prophylaxis, and thromboprophylaxis indications after 24 hours were not examined in this study.

We captured qualitative data on the ease of form implementation and use via formal feedback sessions, as well as paper and electronic questionnaires distributed to healthcare providers at the intervention hospitals at the end of the study.

The primary goal of this pilot trial was to assess the feasibility of implementing the SENTRY intervention. Therefore, the sample size was primarily based on feasibility considerations, not the trial’s secondary outcomes (*i.e.*, rates of appropriate prophylaxis, errors of omission, and errors of commission) [26,27]. To achieve “definite feasibility”, we specified that the baseline proportion of 61% of patients appropriately managed for thromboprophylaxis in the control group (a figure based on previous studies conducted at our institution) would have to increase to 76.25% in the intervention group (Lloyd NS, Douketis JD, Cheng J, Moinuddin I, Pai M, Thabane L, Cook DJ, Schunemann HJ, Spencer FA, Haynes RB: Anticoagulant prophylaxis against venous thromboembolism in hospitalized medical patients: an assessment of current practices and determinants of appropriate and inappropriate prophylaxis management, unpublished) (i.e., a 25% relative increase from 61%). To estimate the difference in rates of appropriate thromboprophylaxis between groups with a 95% confidence interval (CI) and a margin of error of 0.05, we estimated that 644 patients were needed per group. Thus, our goal was to recruit a total of 1,290 patients, with at least 645 in each group. As this was a pilot trial, the goal was to generate crude estimates of feasibility and data to calculate an intraclass correlation coefficient (ICC) for use in future studies. Thus, the sample size in SENTRY was not adjusted for clustering.

### Statistical analysis

Patient and cluster demographics are reported using descriptive statistics. The feasibility outcomes are reported as percentages. The rates of appropriate thromboprophylaxis in the intervention and control group were compared using an odds ratio (OR) with an associated 95% CI. Results were considered statistically significant at alpha = 0.05 (two-sided *p* value). Observations within each participating hospital were assumed more likely to be similar than observations between participating hospitals. A logistic model using the generalized estimating equation (GEE) method was used to account for this clustering effect, incorporating both within-hospital and between-hospital variations. An ICC and variance inflation factor (VIF) were also calculated to assess the impact of the clustering effect [28]. When VIF is >2, the impact of the clustering effect is considered to be large. Quantitative analyses were conducted using SAS 9.3 (SAS Institute Inc., Cary, NC). Qualitative data were summarized in duplicate by two individuals (MP and NL), and comments were grouped into thematic areas.

## Results

**Figure 2:**
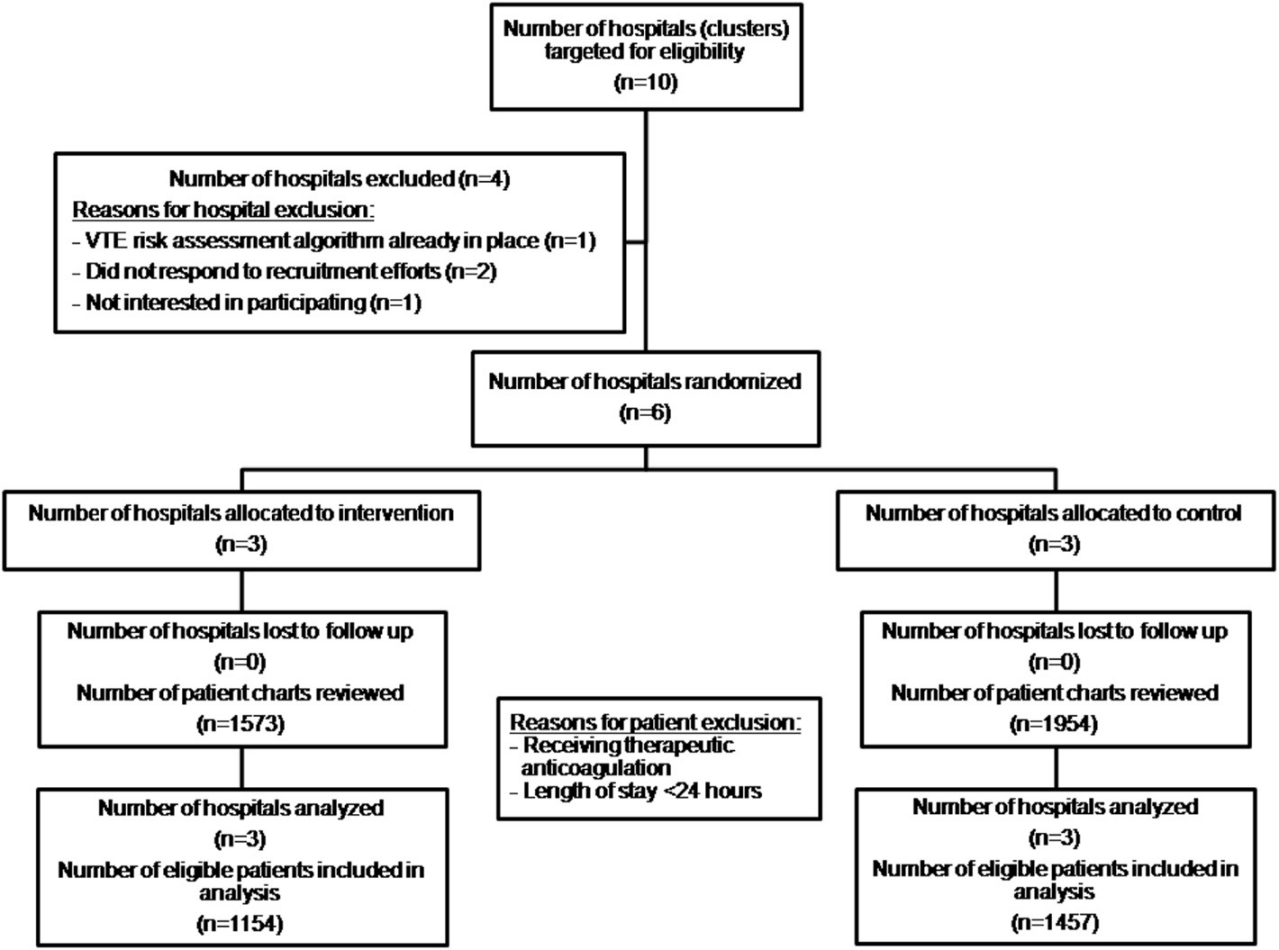
Flow of clusters and participants through SENTRY trial.

Six Ontario hospitals participated in the SENTRY study (see Figure 2). A total of 3,527 medical patient charts were reviewed during the 16-week study period; 2,611 patients (1,154 intervention, 1,457 control) were eligible and included in the analysis. The remaining 916 patients were excluded because they were <18 years of age and/or receiving therapeutic anticoagulation at the time of admission and/or had already been included in the study during a previous admission. Table 2 outlines the demographic characteristics of eligible patients, while Table 3 outlines the demographic characteristics of eligible clusters. The intervention and control groups were comparable. However, some baseline differences were noted. Specifically, there was a higher prevalence of acute respiratory disease (16% versus 9%) and sepsis (13% versus 8%) in the intervention group, and a higher prevalence of cancer in the control group (12% versus 8%). There was a higher prevalence of patients at risk for bleeding in the intervention group (10% versus 6%), as well as a higher prevalence of patients at risk for VTE without risk of bleeding (38% versus 32%). Clusters were comparable for baseline characteristics. Table 4 outlines the prophylaxis options prescribed in the intervention and control group hospitals.

**Table 2:** Patient characteristics

	**Intervention group**	**Control group**
	**(total n = 1154)**	**(total n = 1457)**
**Age (y): Median (min, max)**	72 (18,100)	72 (18,102)
**Male: n (%)**	534 (46)	688 (47)
**Length of stay (days): Median (min, max)**	4 (0,79)	5 (0,133)
**VTE risk factors: n (%)**		
Congestive heart failure	140 (12)	166 (11)
Acute respiratory disease	181 (16)	133 (9)
Acute ischemic stroke	68 (6)	92 (6)
Cancer	93 (8)	172 (12)
Sepsis	145 (13)	111 (8)
Acute neurological disease	24 (2)	13 (1)
Inflammatory bowel disease	23 (2)	25 (2)
Prior VTE	26 (2)	22 (2)
Immobility	637 (55)	806 (55)
**Bleeding risk factors: n (%)**		
Acute bleeding	64 (6)	134 (9)
At risk for bleeding	116 (10)	90 (6)
Recent bleeding	22 (2)	5 (0)
Coagulopathy	93 (8)	185 (13)
Thrombocytopenia	36 (3)	50 (3)
**VTE risk profile: n (%)**		
At risk for VTE without risk of bleeding	436 (38)	465 (32)
At risk for VTE with risk of bleeding	132 (11)	167 (11)
Not at risk for VTE	586 (51)	825 (57)

All percentages use total number of eligible patients as denominator.

VTE = venous thromboembolism.

**Table 3:** Cluster characteristics

	**Intervention group**	**Control group**
	**(total n = 3)**	**(total n = 3)**
Number of eligible patients per cluster: Mean (min, max)	460 (367, 616)	375 (238, 510)
Number of hospitals with a consultative thromboembolism service	1	1
Geographic area	Southern Ontario	Southern Ontario
Number of hospitals in Ontario Ministry of Health and Longterm Care Hospital Class:		
Class A (academic hospitals)	1	1
Class B (community hospitals, >100 beds)	1	1
Class C (community hospitals, <100 beds)	1	1

**Table 4:** Prophylaxis ordered in intervention and control groups

	**Patients in intervention group receiving**	**Patients in control group receiving**
**No prophylaxis**	62%	73%
**Unfractionated heparin at suggested prophylaxis dose**	23%	19%
**Low-molecular-weight heparin at suggested prophylaxis dose**	11%	14%
**Anti-embolic stockings**	6%	2%
**Intermittent pneumatic compression devices**	0%	0%

The VTE risk profiles of patients at intervention and control hospitals were comparable (see Tables 2, 3). At the end of the trial, 64.5% (744/1,154) of patients in the intervention group and 66.6% (970/1,457) of patients in the control group were appropriately managed for thromboprophylaxis within 24 hours of admission. After adjusting for within-hospital correlation, there was no significant difference between the rates of appropriate thromboprophylaxis between groups (OR = 0.80 in intervention versus control group; 95% CI: 0.50, 1.28; *p* = 0.36 after adjustment for clustering effect; ICC = 0.022; VIF = 10.55; see Table 5). The rates of errors of commission and errors of omission were not significantly different between groups. (Errors of omission: OR = 1.30 in intervention versus control group; 95% CI: 0.68, 2.50; *p* = 0.43, after adjusting for clustering effect. Errors of commission: OR = 1.01 in intervention versus control group; 95% CI: 0.43, 2.37; *p* = 0.97, after adjusting for clustering effect.) The intervention did not achieve the prespecified target of definite feasibility, defined as a rate of appropriate thromboprophylaxis of 76% in the intervention group.

**Table 5:** Thromboprophylaxis in intervention and control groups

	**Intervention group, n (%)**	**Control group, n (%)**	**Odds ratio (95% confidence interval), *p *value**
**Number of patient charts reviewed**	1573	1954	
**Eligible patients**	1154 (73)	1457 (75)	
**Received appropriate prophylaxis strategy**	744 (64)	970 (67)	0.80 (0.50, 1.28), *p* = 0.36
**Appropriate receipt of prophylaxis**	263 (23)	290 (20)	1.09 (0.39, 3.11), *p* = 0.86
**Appropriate nonreceipt of prophylaxis**	481 (42)	680 (47)	0.76 (0.40, 1.44), *p* = 0.40
**Received inappropriate prophylaxis strategy**	410 (33)	487 (33)	1.25 (0.78, 1.99), *p* = 0.36
**Error of commission (inappropriate receipt of prophylaxis)**	105 (9)	145 (10)	1.01 (0.43, 2.37), *p* = 0.97
**Error of omission (inappropriate nonreceipt of prophylaxis)**	305 (26)	342 (23)	1.30 (0.68, 2.50), *p* = 0.43

All percentages use total number of eligible patients as denominator. All odds ratios compare the specified group to all other eligible patients.

Feedback was received from 39 completed questionnaires (21 physicians, 10 nurses, 8 pharmacists) out of over 100 questionnaires distributed to healthcare providers at the intervention hospitals. Feedback was also received via the formal feedback sessions. Four themes were identified: order form accessibility, order form content, factors that reinforced appropriate prophylaxis, and the reason why inappropriate prophylaxis rates remained high. Physicians stated that forms were sometimes inaccessible and that forms needed to be available in the emergency department, where patients were generally first assessed by the medicine service. Nurses stated that they often forgot to put the form on patients’ charts or prompt physicians to use it. They attributed this to many other forms competing for their attention. Many respondents suggested that if the forms were automatically placed on every chart as part of a standard admission protocol, uptake would be improved. The content of the form received mixed reviews, and many physicians emphasized that the ACCP guidelines (which formed the basis of the risk assessment algorithm) were not “one size fits all.” They stated that general medical patients frequently presented exceptions to the rules in the risk assessment algorithm. Respondents also stated that some of the terms in the algorithm, such as “immobility” and “sepsis”, needed to be more clearly defined. Participants reported that educational sessions reinforced their use of appropriate prophylaxis, though their busy schedules and lack of protected educational time made it difficult to attend these sessions. They also suggested that negative reinforcement, such as practitioner-specific report cards citing specific errors, might be more effective than reporting aggregate data. Finally, when asked why inappropriate prophylaxis rates remained high, each group of healthcare professionals—physicians, nurses, and pharmacists—considered the other groups to be responsible for the inappropriate management. They expressed that their own workload was too heavy to take on additional primary responsibility for thromboprophylaxis and that other groups should take more ownership of the problem.

## Discussion

Hospitals that were allocated to receive our multicomponent intervention comprising education, standardized paper-based physician orders, and group audit and feedback did not have a higher rate of hospitalized medical patients appropriately managed for thromboprophylaxis within 24 hours of admission than did hospitals that were not allocated to this strategy (63% vs. 67%). This finding, coupled with the problems associated with ensuring preprinted orders were placed in all medical charts, led us to conclude that this intervention should not be provided on a larger scale without major revision and testing. That is, it was not feasible. The study was primarily designed to assess the feasibility of a future large cluster randomized trial. It was conducted in a broad spectrum of university and community-based hospital settings. Thus, the feasibility assessments and results are generalizable to similar settings.

Several observations may explain our findings. First, despite our efforts to encourage the use of the thromboprophylaxis forms for every admitted patient, staff stated that they often forgot to use them, as they were overwhelmed by the many other forms in circulation on the wards. This suggests that for an order set to be successful, it may have to be embedded within a mandatory, widely available patient admission package. Giving healthcare providers an opportunity to opt out or overlook an order set can result in poor compliance. Second, though the SENTRY order set was modeled on the widely accepted ACCP guidelines for thromboprophylaxis, clinicians stated that it was cumbersome and not applicable to every clinical situation. Thromboprophylaxis guidelines for general medical patients are clearly not universally accepted. The ACCP guidelines are all “strong” recommendations, based on high-quality evidence (RCTs without important limitations or strong evidence from observational studies) [29]. However, hospitalized medical patients are complex and heterogeneous. “One size fits all” guidelines may not translate well to this patient population, and the clinical gestalt may play an important role in risk stratification. As suggested by the ACCP guideline authors themselves, the feasibility, usability, practicality, and applicability of even well-supported recommendations may differ in specific subgroups of patients [30]. Clinicians may rightly refrain from implementing thromboprophylaxis guidelines in certain patients, based on their specific characteristics. Thus, an important process measure to consider in future studies would be whether thromboprophylaxis was *considered* in every patient, not just if it followed the guidelines. Finally, despite efforts to engage all members of the healthcare team, there was a lack of awareness of the SENTRY trial in the intervention hospitals during its conduct. (By design, awareness was not measured in the control hospitals where practice was unchanged.) Busy clinical schedules and “information overload” prevented staff from attending educational sessions and reviewing posters and leaflets. There was also a lack of involvement of clinical and administrative leaders, resulting in the absence of a sustained internal push to improve practice. Recruiting a single local PI at each intervention site did not promote the use of the standardized forms and consideration of thromboprophylaxis in every patient. This intervention may have been more successful had there been buy-in and promotion of this patient safety initiative by the majority of staff physicians on the medical service.

When compared to the impact of other KT studies of thromboprophylaxis, the lack of comparable success achieved by SENTRY can be explained by two additional considerations. All prior low-tech studies (and all prior high-tech studies apart from Galanter *et al.*) were conducted at a single center. Our trial was unique, as we implemented our intervention in several centers across the province, to assess its generalizability. However, when we attempted to “scale up” our KT intervention, the increased demand on time and resources proved to be challenging. Previous single-center studies examined geographically contained systems [15,17-20]. It may be easier to effect change in these closed systems, as there is a concentration of resources, a single target audience, and recognized opinion leaders. In addition, many of the most successful studies incorporated high-tech elements, such as computerized decision support systems (CDSS) and electronic alerts [15-18]. Our region, like many others, does not have the resources or infrastructure to adopt high-tech CDSSs or electronic alerts. There is widespread enthusiasm for CDSSs in part because it is perceived that they require less clinical decision making than order sets and can guide healthcare providers towards evidence-based practices. Though there is some evidence that CDSSs can improve practitioner performance, further research is needed to determine their effect on patient-important outcomes and their cost-effectiveness [31].

Our study has several potential limitations that may have impacted the feasibility of the intervention. First, as many patients are admitted to the medical service from the emergency department, we recognize that uptake of the forms may have increased had they been available in the ER as well, instead of solely on the medical wards. Future studies require intervention at all points of entry to the inpatient service. Second, though all information was audited from patients’ charts, some variables (*e.g.*, immobility) were ambiguously defined and inconsistently documented by healthcare providers. This may have resulted in an incomplete picture of patients’ thrombosis and bleeding risks. Third, physicians in this study rarely documented their reasoning if they inappropriately prescribed or omitted prophylaxis. Study participants told us that they often used clinical judgment as an adjunct to guidelines when prescribing thromboprophylaxis, but the lack of documentation did not allow us to fully explore the impact of clinical judgment on prescribing practices. Fourth, the house staff at the two academic centers in the study may have been a source of contamination. Additional house staff occasionally provided overnight coverage at the intervention group academic center. These additional house staff were not formally educated about the study, so they effectively functioned as if they were in the control group. Conversely, additional house staff who provided overnight coverage at the control group academic center may have been previously educated about our study while working at the intervention group academic center. Thus, they effectively functioned as if they were in the intervention group. The resulting bias is somewhat offset by the fact that staff physicians did not rotate between sites and that their clinical judgment ultimately guided patient care. Fifth, the strength of our study is compromised by the lack of data on fidelity in the intervention group (*i.e.*, the extent to which the intervention components were used/received as intended). For example, we did not formally collect data on the level of uptake of the forms at each site, or the attendance rates at the educational sessions. These data would have allowed us to more thoroughly investigate the feasibility of the KT intervention and monitor for potential contamination. Sixth, our study was not designed to measure safety and efficacy outcomes of thromboprophylaxis, such as thrombosis, heparin-induced thrombocytopenia, or major bleeding. Seventh, we did not measure baseline data on appropriate thromboprophylaxis rates at each hospital prior to implementing the intervention. Instead, we used previously reported rates. Had we obtained baseline rates from the study sites, we may have seen a significant difference in the secondary outcomes, as we would have been comparing improvement within each site. Eighth, it is possible that baseline differences in the intervention and control groups reduced the feasibility of our intervention. There was a higher prevalence of patients at risk for VTE (without risk of bleeding) in the intervention group. Though the preprinted orders accounted for patients with a spectrum of bleeding and thrombotic risks, it is possible that clinicians in the intervention hospitals were not comfortable applying an algorithmic VTE prophylaxis approach to "sicker" patients (though their illnesses contributed to an increased risk of VTE). Finally, we did not adjust the sample size in this study for clustering. This was intentional; SENTRY, like other pilot trials, was designed to generate crude estimates of intervention feasibility and sufficient data to calculate an ICC for use in future studies. Analysis of the secondary outcomes was exploratory and should be judged on the basis of feasibility results.

The strengths of our study include participation of both academic and community hospitals, screening of all consecutive medical admissions during the study period, and the use of a standardized KT intervention and data collection form. We also used rigorous operational definitions to determine whether each patient’s thromboprophylaxis management was “appropriate”. Though many previous studies have reported rates of thromboprophylaxis, we went beyond this outcome, and aimed to determine whether prophylaxis was appropriate or not using the widely accepted 8^th^ ACCP Evidence-Based Clinical Practice Guidelines on Antithrombotic Therapy. This *a priori* decision to consider not only omission or commission of prophylaxis, but to consider evidence-based management as well, is another strength of our study. Finally, acknowledging that quantitative outcomes are insufficient to explain why an intervention is successful or not, we also captured qualitative data to elaborate on the impact of our intervention.

The key lesson learned from this study was that a KT intervention that relies on the voluntary cooperation of front-line clinicians (*i.e.*, a bottom-up approach) was not feasible, and thus insufficient to effect change in thromboprophylaxis patterns. While we still advocate a front-line team approach to thromboprophylaxis, a top-down push from clinical and administrative leaders may also be necessary [32-34]. Although our prior qualitative study did not support the contention that quality improvement is a shared responsibility among different clinician groups [21], accreditation standards today are such that both clinicians and managers are responsible for patient safety. Thus, clear support for thromboprophylaxis at the level of the healthcare organization may address some very specific modifiable barriers to change identified by SENTRY participants, including the lack of protected time for team members to participate in education and the nonmandatory use of quality improvement resources. At the healthcare system level, the consideration of thromboprophylaxis appropriate to the individual patient as a performance measure, tied to healthcare accreditation and remuneration, may also cultivate a culture of patient safety, reinforce evidence-based practice, and reward centers for positive results. This study identified several factors that may increase uptake of a VTE prophylaxis strategy, including local champions, support from clinical and administrative leaders, mandatory use, and a simple, clinically relevant risk assessment tool. We are currently undertaking a controlled trial of electronic order entry and a CDSS, which may also facilitate appropriate prophylaxis. The effectiveness of these broader strategies on patient and provider outcomes must be determined by further high-quality research.

## Competing interests

The authors declare no competing interests.

## Authors’ contributions

MP and NSL were responsible for study design and implementation, data analysis, and writing the paper. JC and LT provided statistical consultation, were responsible for data analysis, and contributed to writing the paper. FAS, DJC, RBH, LT, HJS, and JDD contributed to study conception and design, data analysis, and writing the paper. All authors read and approved the final manuscript.
